# Risk factors for postoperative delirium in geriatric patients with hip fracture: A systematic review and meta-analysis

**DOI:** 10.3389/fnagi.2022.960364

**Published:** 2022-08-03

**Authors:** Yi-ming Qi, Ying-juan Li, Ji-hong Zou, Xiao-dong Qiu, Jie Sun, Yun-feng Rui

**Affiliations:** ^1^Department of Orthopaedics, School of Medicine, Zhongda Hospital, Southeast University, Nanjing, China; ^2^Multidisciplinary Team (MDT) for Geriatric Hip Fracture Comprehensive Management, School of Medicine, Zhongda Hospital, Southeast University, Nanjing, China; ^3^Orthopaedic Trauma Institute, Southeast University, Nanjing, China; ^4^School of Medicine, Southeast University, Nanjing, China; ^5^Department of Geriatrics, School of Medicine, Zhongda Hospital, Southeast University, Nanjing, China; ^6^Department of Anesthesiology, School of Medicine, Zhongda Hospital, Southeast University, Nanjing, China; ^7^Trauma Center, Zhongda Hospital, Southeast University, Nanjing, China

**Keywords:** risk factors, postoperative delirium, geriatric, hip fracture, systematic review, meta-analysis

## Abstract

**Objectives:**

This systematic review and meta-analysis was conducted to identify the potential risk factors for postoperative delirium in geriatric patients with hip fracture.

**Methods:**

PubMed, EMBASE, and Cochrane Library were searched from inception until December 31st, 2021. A combined searching strategy of subject words and free words was adopted. Studies involving risk factors for postoperative delirium in elderly patients undergoing hip fracture surgeries were reviewed. Qualities of included studies were assessed using the Newcastle–Ottawa Scale. Data were pooled and a meta-analysis was performed using Review Manager 5.3.

**Results:**

A total of 37 studies were included. The following risk factors were significant: advanced age (per year increase) (OR: 1.05, 95% CI 1.04–1.07), age>80 years (OR: 2.26, 95% CI 1.47–3.47), male (OR: 1.53, 95% CI 1.37–1.70), preoperative cognitive impairment (OR:3.20, 95% CI 2.12–4.83), preoperative dementia (OR: 2.74, 95% CI 2.18–3.45), preoperative delirium (OR: 9.23, 95% CI 8.26–10.32), diabetes (OR: 1.18, 95% CI 1.05–1.33), preoperative functional dependence (OR: 1.31, 95% CI 1.11–1.56), ASA level (per level increase) (OR: 1.63, 95% CI 1.04–2.57), ASA level≥3(OR: 1.76, 95% CI 1.39–2.24), low albumin (OR: 3.30, 95% CI 1.44–7.55), medical comorbidities (OR: 1.15, 95% CI 1.06–1.25), Parkinson's disease (OR: 4.17, 95% CI 1.68–10.31) and surgery delay>48 h (OR: 1.90, 95% CI 1.36–2.65).

**Conclusions:**

Clinicians should be alert to patients with those risk factors. To identify the risk factors more precisely, more research studies with larger sample size and better design should be conducted.

## Introduction

The incidence of hip fracture is increasing concurrently with the aging of the population. It has been estimated that in many countries, the number of hip fractures will rise from 1.7 million in 1990 to 6.3 million in 2050 (Gullberg et al., 1997). One of the complications associated with hip fracture is postoperative delirium.

Delirium is a common neuropsychiatric syndrome that can happen in hospitalized patients from different settings. It has three notable characteristics: acute onset of altered mental status, difficulty in sustaining attention or changing attention and a fluctuating course (Greer et al., 2011). In surgical departments, the incidence is particularly high, especially in geriatric patients undergoing surgery with hip fracture, where the prevalence can reach as high as around 50% (Goldenberg et al., 2006; Shin et al., 2016; Jeon and Sohng, 2021). Significant negative consequences concerned about postoperative delirium in hip fracture patients include higher postoperative complications, poorer functional recovery, more readmissions and reoperations and even higher mortality (Haynes et al., 2021; Jeon and Sohng, 2021). The good news is that delirium is referred to be preventable in about 40% of patients, which makes it meaningful and attractive to take proactive measures to prevent the process of delirium (None, 2015). Given these negative consequences, the high incidence and preventability of delirium following hip fracture surgery in this population, the identification of those patients at risk of postoperative delirium is of great significance.

Risk factors in terms of delirium after hip fracture surgeries have been researched in many studies, while they have not reached an agreement (Kim et al., 2020; Wang et al., 2021; Ahn and Bang, 2022). Former meta-analysis have explored some potential risk factors for delirium in hip fracture patients (Yang et al., 2017; Wu et al., 2021), however, the patients in some included studies are not all geriatric patients, and the risk factors reported in some included studies are not merely for postoperative delirium, but for preoperative or perioperative delirium. Besides, many articles have been published after those meta-analysis were published, which may provide some new evidences for or against previous outcomes. Therefore, this meta-analysis was conducted to identify the potential risk factors for postoperative delirium in geriatric in patients with hip fracture.

## Methods

This meta-analysis is conducted according to the Preferred Reporting Items for systematic Reviews and Meta-Analyses (PRISMA) Statement (Moher et al., 2009).

### Search strategy

PubMed, EMBASE, and Cochrane Library were searched from inception until December 31st, 2021. A combined searching strategy of subject words and free words was adopted. The concrete searching strategy for PUBMED is as follows:(“femur neck fractures”[Title/Abstract] OR “fractures, femur neck”[Title/Abstract] OR “fractures, femoral neck”[Title/Abstract] OR “Femoral neck fractures”[Title/Abstract] OR “fractures, subtrochanteric”[Title/Abstract] OR “subtrochanteric fractures”[Title/Abstract] OR “fractures, intertrochanteric”[Title/Abstract] OR “intertrochanteric fractures”[Title/Abstract] OR “fractures, trochanteric”[Title/Abstract] OR “trochanteric fractures”[Title/Abstract] OR “fractures, pertrochanteric”[Title/Abstract] OR “pertrochanteric fractures”[Title/Abstract] OR “fractures, hip”[Title/Abstract] OR “Hip Fractures”[Title/Abstract] OR “hip surgery”[Title/Abstract] OR “Femoral neck fractures”[MeSH Terms] OR “Hip Fractures”[MeSH Terms]) AND (“Risk Factors”[MeSH Terms] OR [“correlat^*^”[Title/Abstract] OR “factor^*^”[Title/Abstract] OR “risk”[Title/Abstract] OR “predict^*^”[Title/Abstract])] AND (“Delirium”[MeSH Terms] OR [“deliri^*^”[Title/Abstract] OR “confus^*^”[Title/Abstract] OR “pocd”[Title/Abstract] OR “postoperative cognitive disorder”[Title/Abstract])].

### Eligibility criteria

The inclusion criteria were as follows: (1) Types of studies: retrospective or prospective cohort design; (2) Types of participants: all the patients are older than 60 years old and undergo surgeries for hip fracture; (3) Outcomes: risk factors merely for postoperative delirium, and the risk factors are reported in ≥2 studies. (4) Data: available odds ratio (OR) or relative risk (RR) with 95% confidence interval (95% CI) as a result of a multivariate logistic regression.

The exclusion criteria were as follows: (1) Types of studies: those studies that are not cohort design or whose concrete description could not be extracted, editorial reviews, systematic reviews, conference abstracts, letters and comments; (2) Types of participants: including patients younger than 60 years old or undergoing other types of surgeries; (3) Outcomes: assessed risk factors for postoperative delirium are reported in less than 2 studies or risk factors for preoperative delirium or perioperative delirium; (4) Data: no available odds ratio (OR) or relative risk (RR) with 95% confidence interval (95% CI) as a result of a multivariate logistic regression.

### Data extraction and quality assessment

Two reviewers independently scanned the titles and abstracts of potentially included studies. Once the studies met the inclusion criteria, full texts of articles were reviewed thoroughly. The following variables were extracted from each study: first author's name, publication year, country, diagnosis of delirium and numbers of cases and controls, mean age of cases and controls, numbers of males and females in cases and controls, and significant risk factors. The extracted data were entered in a standardized data collection form. Any discrepancy about the data were resolved by discussion or consulting a senior reviewer.

The quality of included studies was assessed by 2 reviewers with the Newcastle–Ottawa Scale (NOS) (Wells et al., 2014) based on the three main items: the selection of the study groups (0–4 points), the comparability of the groups (0–2 points) and the determination of either the exposure or the outcome of interest (0–3 points). The NOS score ranges from 0 to 9. A study with a score more than 7 was considered to be of high quality.

### Statistical analysis

The meta-analysis was conducted with the Review Manager 5.3 software (The Cochrane Collaboration, Oxford, UK). Odds ratios (ORs) and 95% confidence intervals (CIs) were pooled across the studies to estimate the risk factors of postoperative delirium. In studies where the incidence of delirium is low, the RR could be regarded as equal to the OR. Statistical heterogeneity was assessed with the P and I^2^ values using the standard Chi-square test. When I^2^>50%, or *P* < 0.1, significant heterogeneity was indicated and a random-effects model was applied for the meta-analysis. Otherwise, a fixed-effects model was used. To assess the publication bias, a funnel plot of the primary outcome will be utilized. When it is necessary, sensitive analysis will be conducted by excluding outlier studies one by one to verify the source of heterogeneity.

## Results

**Figure 1:**
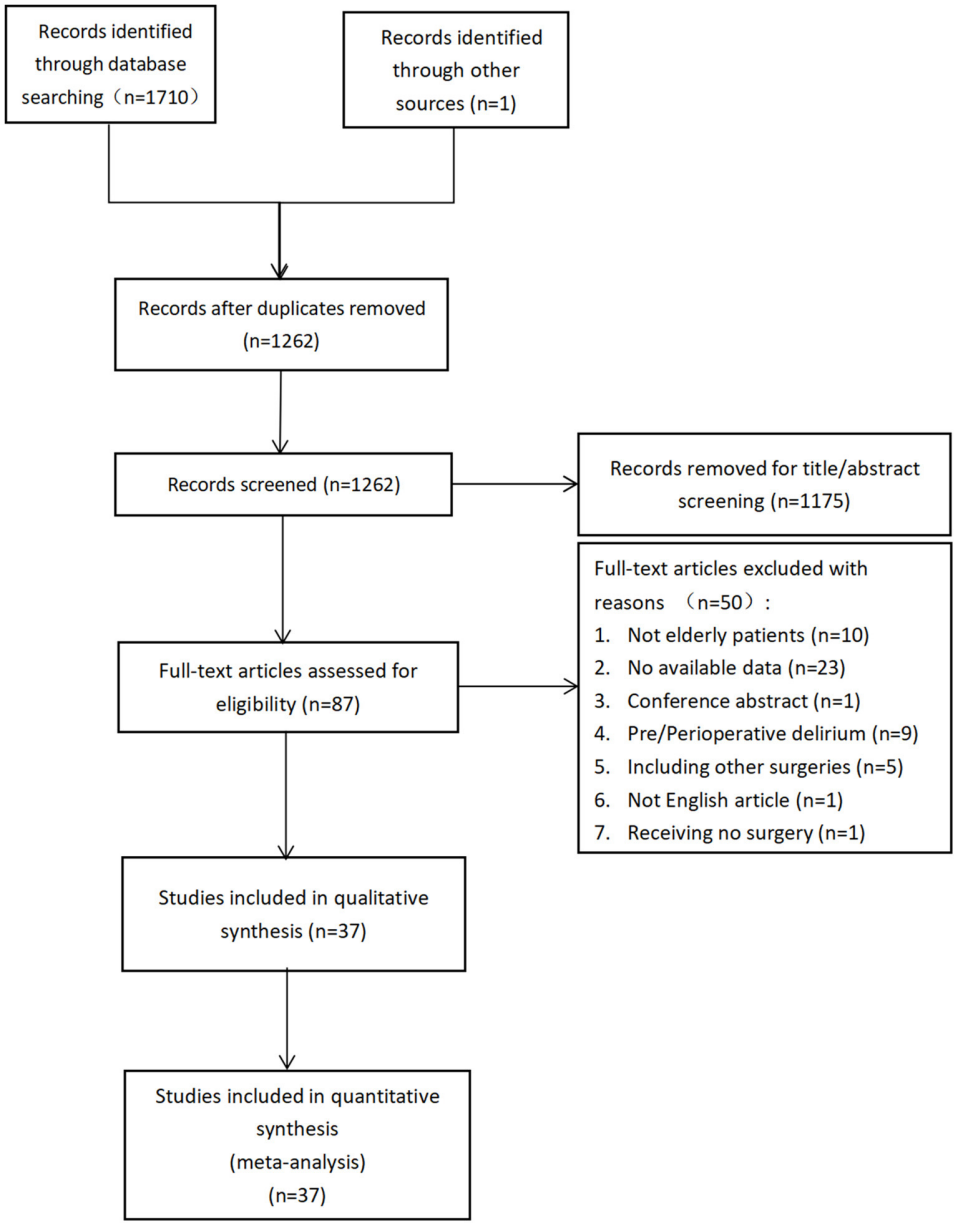
The flow diagram of the search process of the literature and the result of the literature search.

One thousand seven hundred ten articles were identified from the search of the databases and 1 paper was identified from other sources. One thousand two hundred sixty-two studies remained when the duplicates were removed. Then, the title and the abstracts of the 1262 citations were scanned to exclude those which did not meet the inclusion criteria. As a consequence, 1175 citations were excluded. Next, the 87 remained studies were carefully full-text-reviewed to recognize those which could reach the inclusion criteria. At last 37 citations (Kagansky et al., 2004; Goldenberg et al., 2006; Juliebø et al., 2009; Chrispal et al., 2010; Lee et al., 2011; Vochteloo et al., 2011; Nie et al., 2012; Kim et al., 2013, 2020; Chen et al., 2014; Guo et al., 2016; Oh et al., 2016; Shin et al., 2016; van der Zanden et al., 2016; Zheng et al., 2016; Choi et al., 2017; Koskderelioglu et al., 2017; Mazzola et al., 2017; Arshi et al., 2018; Flikweert et al., 2018; Levinoff et al., 2018; Wang et al., 2018, 2021; Agrawal et al., 2019; Harris et al., 2019; Ravi et al., 2019; Zhang et al., 2019; Aldwikat et al., 2020; Cho et al., 2020; He et al., 2020; Uzoigwe et al., 2020; Xing et al., 2020; Davani et al., 2021; Haynes et al., 2021; Jeon and Sohng, 2021; Oberai et al., 2021; Ahn and Bang, 2022) were included for further qualitative and quantitative synthesis (Figure 1).

### The general characteristics of the included studies

In the included 37 studies, 17 studies were conducted in Asia (9 in China, 6 in Korea, 1 in India and 1 in Israel), 11 studies were conducted in North America (9 in the USA and 2 in Canada), 7 studies were conducted in Europe (3 in Netherlands, 1 in UK, 1 in Italy, 1 in Turkey and 1 in Norway) and the remaining 2 were conducted in Australia and New Zealand. The Confusion Assessment Scale (CAM) was the most frequently used scale (in 20 studies) for the diagnosis of postoperative delirium in hip fracture patients. The incidence of postoperative delirium varies among the range from 10.09% to 51.28%. In eight studies, the mean age in delirium patients an non-delirium patients are both older than 80, and in almost all of the studies, female patients are more than male patients in both groups (Additional File 1 in Supplementary material).

### Methodological quality assessment

The result of methodological quality assessment of the included studies are as follows: 20 studies scored 8 points, 13 studies scored 7 points and 4 studies scored 6 points (Additional File 2 in Supplementary material).

### Outcomes for meta-analysis

#### Age and gender

**Figure 2:**
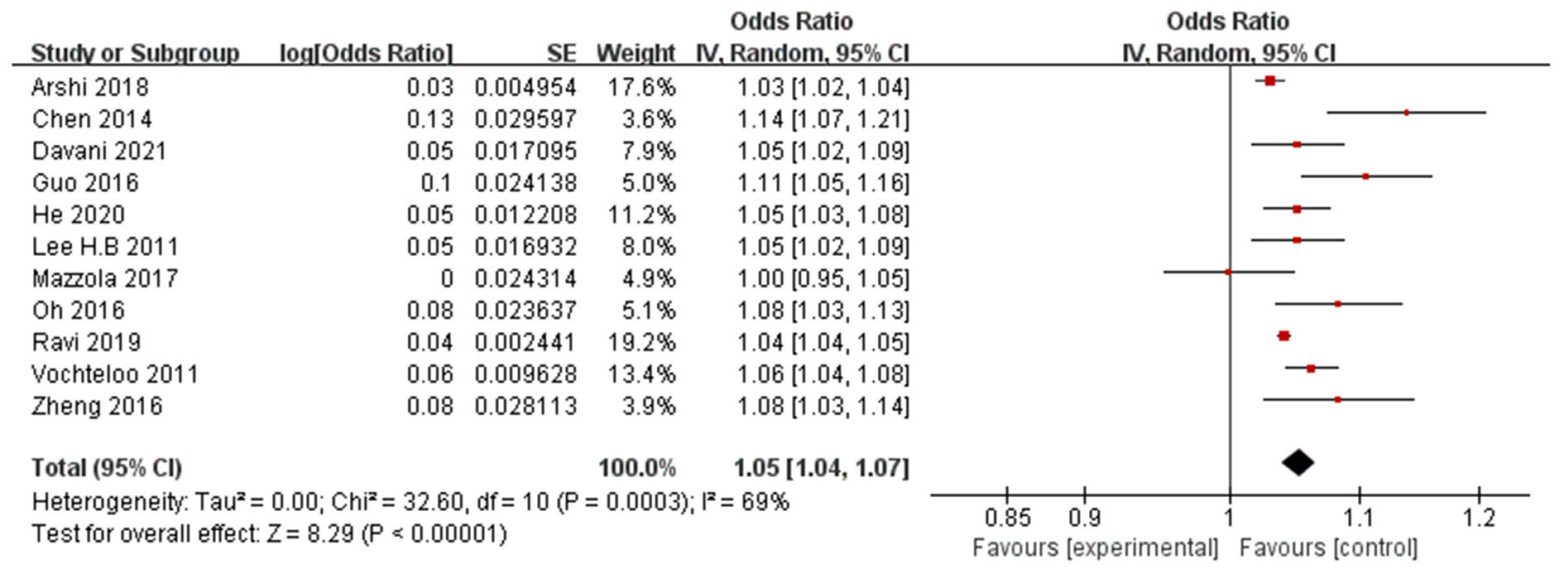
Forest plot for age (per year increase).

**Figure 3:**
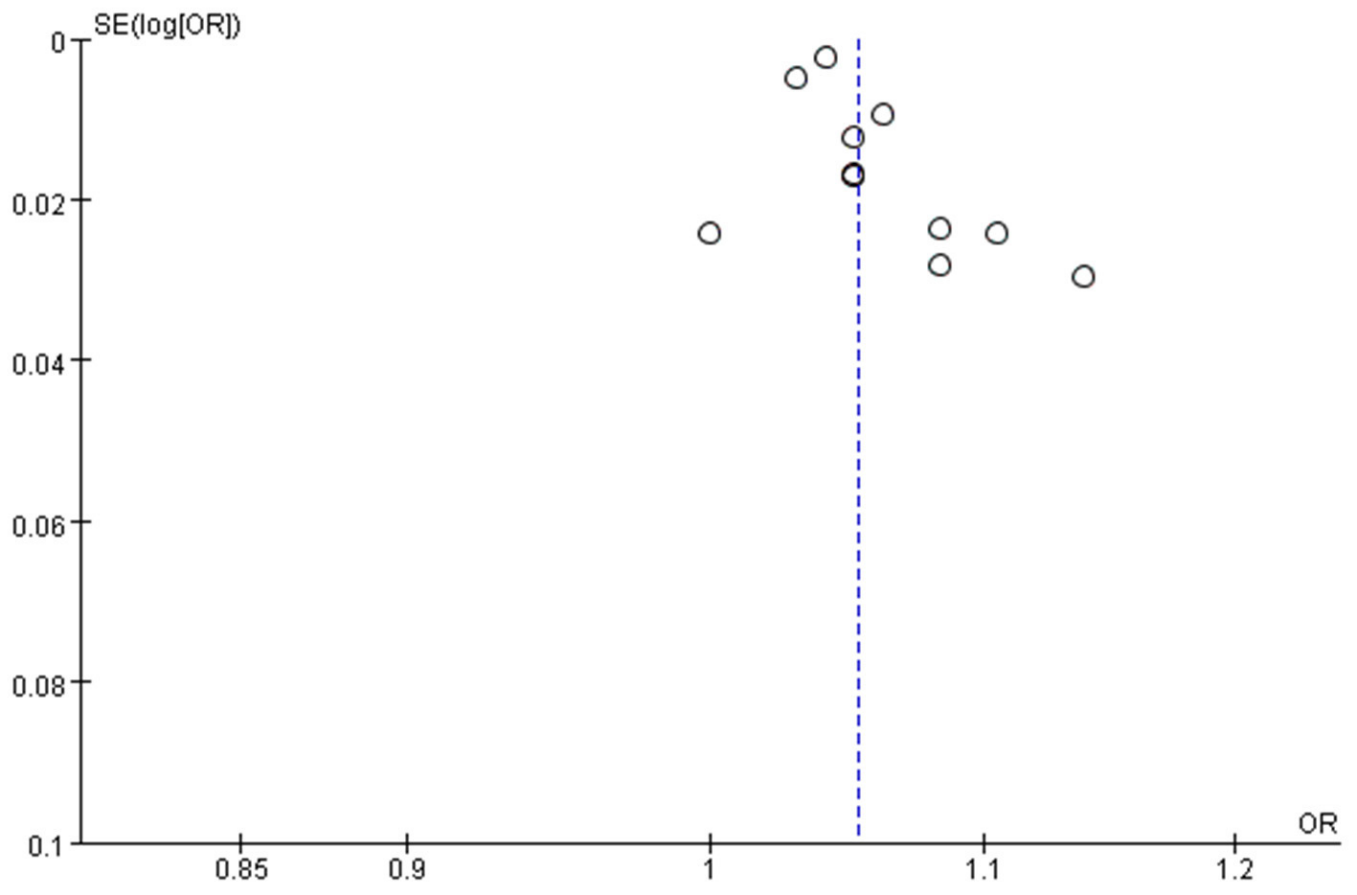
Funnel plot for age (per year increase) for the investigation of publication bias.

**Figure 4:**
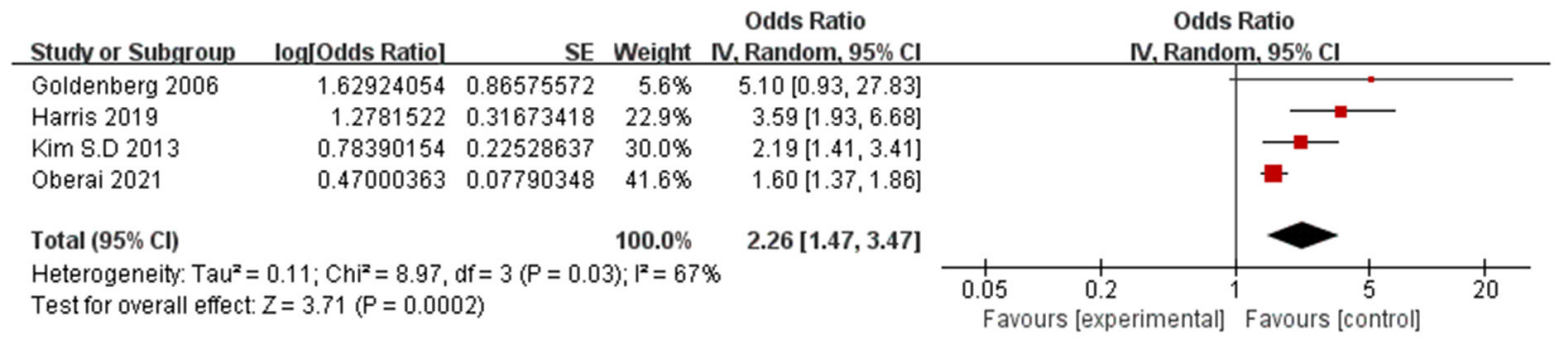
Forest plot for age>80 years.

Eleven papers (Lee et al., 2011; Vochteloo et al., 2011; Chen et al., 2014; Guo et al., 2016; Oh et al., 2016; Zheng et al., 2016; Mazzola et al., 2017; Arshi et al., 2018; Ravi et al., 2019; He et al., 2020; Davani et al., 2021) reported advanced age (per year increase) as a significant risk factor for POD. A random-effects model was applied because significant heterogeneity was found among these studies (*P* = 0.0003, I^2^ = 69%). The meta-analysis of the pooled studies suggest that advanced age (per year increase) is a significant risk factor for the development of POD in geriatric patients after hip fracture surgeries (OR: 1.05, 95% CI 1.04–1.07, *P* < 0.00001, Figure 2). The funnel plot for age (per year increase) was employed to evaluate publication bias. The funnel plot shows that all the dots are generally symmetrically distributed on both sides of the dotted line, which suggests little publication bias for the meta-analysis of advanced age (Figure 3). Another 4 studies (Goldenberg et al., 2006; Kim et al., 2013; Harris et al., 2019; Oberai et al., 2021) reported age>80 years as a significant risk factor for POD. A random-effects model was applied because significant heterogeneity was found among these studies (*P* = 0.03, I^2^ = 67%). The meta-analysis of the pooled studies suggests that age>80 years is a significant risk factor for the development of POD in geriatric patients after hip fracture surgeries (OR: 2.26, 95% CI 1.47–3.47, *P* = 0.0002, Figure 4). This means that the incidence of POD in patients older than 80 years Is 2.26 times the incidence of POD in patients younger than 80 years.

**Figure 5:**
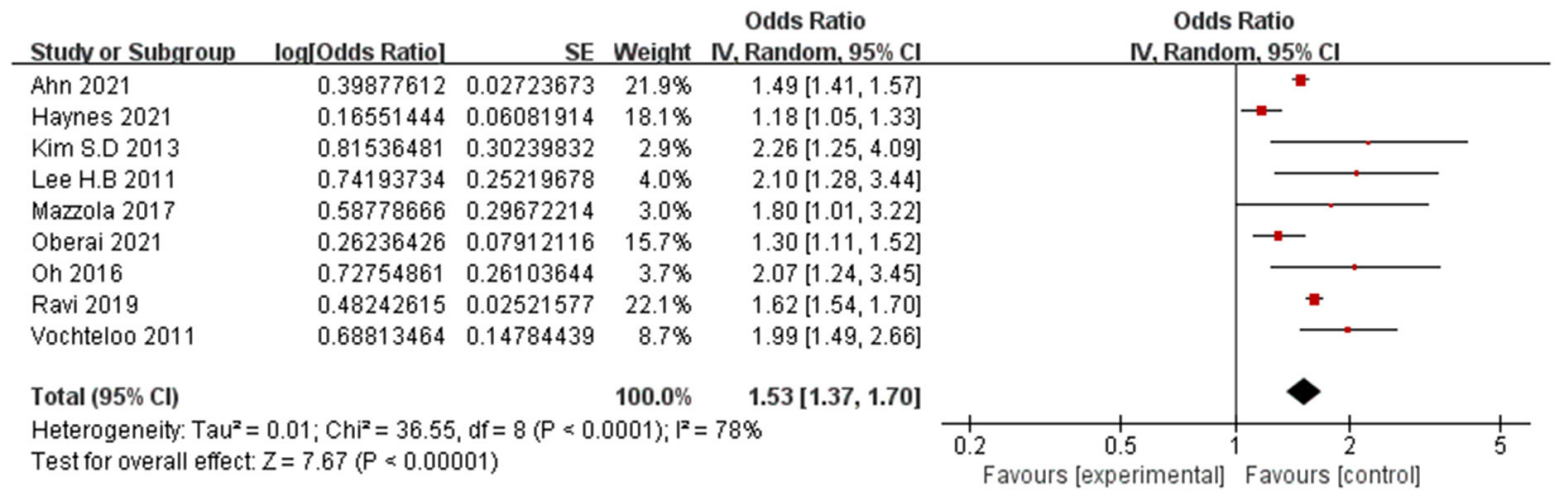
Forest plot for male.

9 Papers (Lee et al., 2011; Vochteloo et al., 2011; Kim et al., 2013; Oh et al., 2016; Mazzola et al., 2017; Ravi et al., 2019; Haynes et al., 2021; Oberai et al., 2021; Ahn and Bang, 2022) reported male as a significant risk factor for POD. A random-effects model was applied because significant heterogeneity was found among these studies (*P* < 0.0001, I^2^ = 78%). The meta-analysis of the pooled studies suggests that male is a significant risk factor for the development of POD (OR: 1.53, 95% CI 1.37–1.70, *P* < 0.00001, Figure 5).

#### Preoperative cognitive impairment

**Figure 6:**
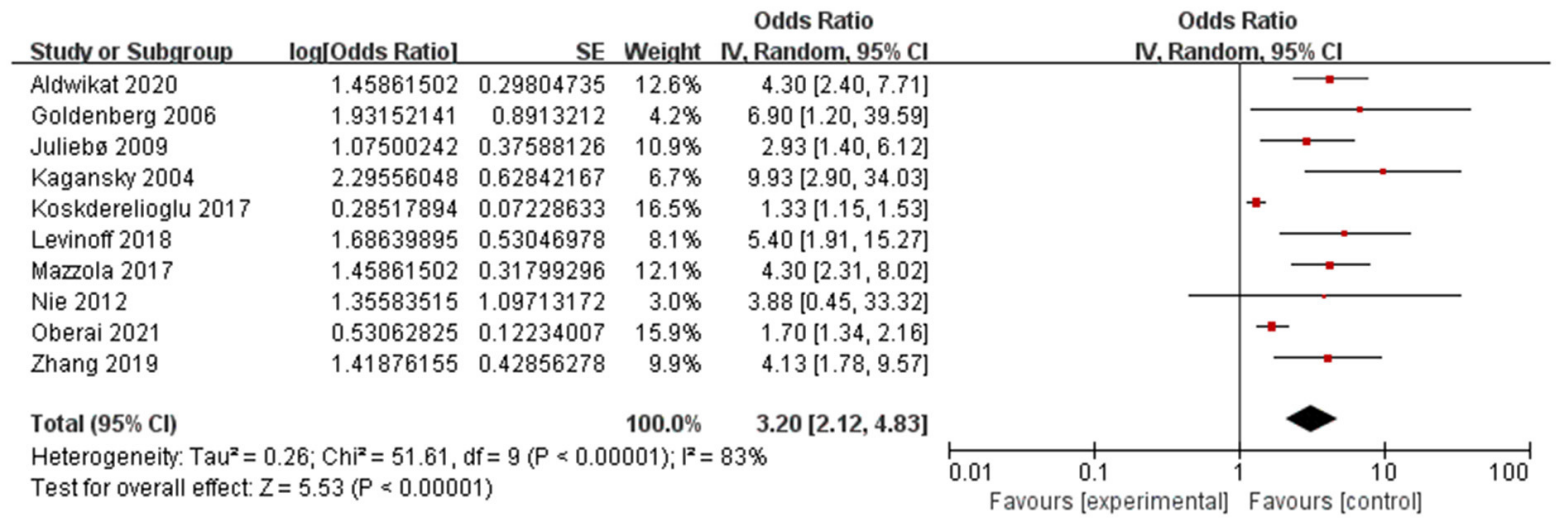
Forest plot for preoperative cognitive impairment.

Ten papers (Kagansky et al., 2004; Goldenberg et al., 2006; Juliebø et al., 2009; Nie et al., 2012; Koskderelioglu et al., 2017; Mazzola et al., 2017; Levinoff et al., 2018; Zhang et al., 2019; Aldwikat et al., 2020; Oberai et al., 2021) reported preoperative cognitive impairment as a significant risk factor for POD. A random-effects model was applied because significant heterogeneity was found among these studies (*P* < 0.00001, I^2^ = 83%). The meta-analysis of the pooled studies suggest that preoperative cognitive impairment is a significant risk factor for the development of POD (OR: 3.20, 95% CI 2.12–4.83, *P* < 0.00001, Figure 6).

#### Preoperative dementia

**Figure 7:**
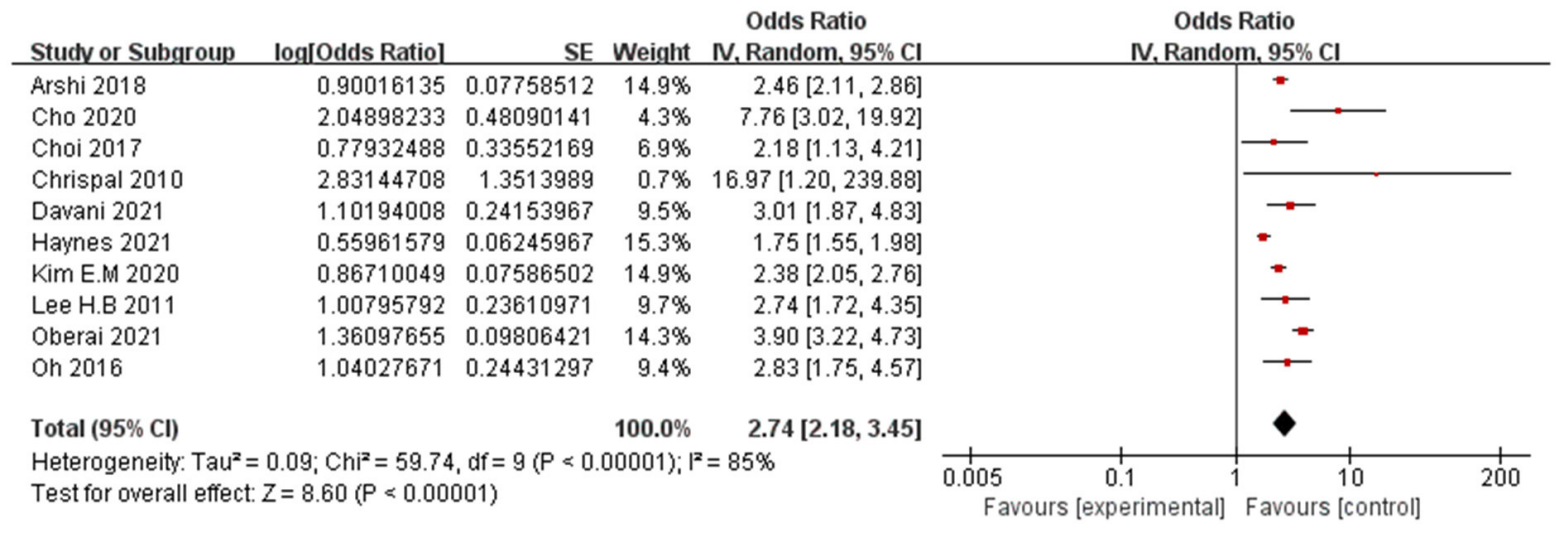
Forest plot for preoperative dementia.

Ten papers (Chrispal et al., 2010; Lee et al., 2011; Oh et al., 2016; Choi et al., 2017; Arshi et al., 2018; Cho et al., 2020; Kim et al., 2020; Davani et al., 2021; Haynes et al., 2021; Oberai et al., 2021) reported preoperative dementia as a significant risk factor for POD. A random-effects model was applied because significant heterogeneity was found among these studies (*P* < 0.00001, I^2^ = 85%). The meta-analysis of the pooled studies suggest that preoperative dementia is a significant risk factor for the development of POD (OR: 2.74, 95% CI 2.18–3.45, *P* < 0.00001, Figure 7).

#### Preoperative delirium

**Figure 8:**
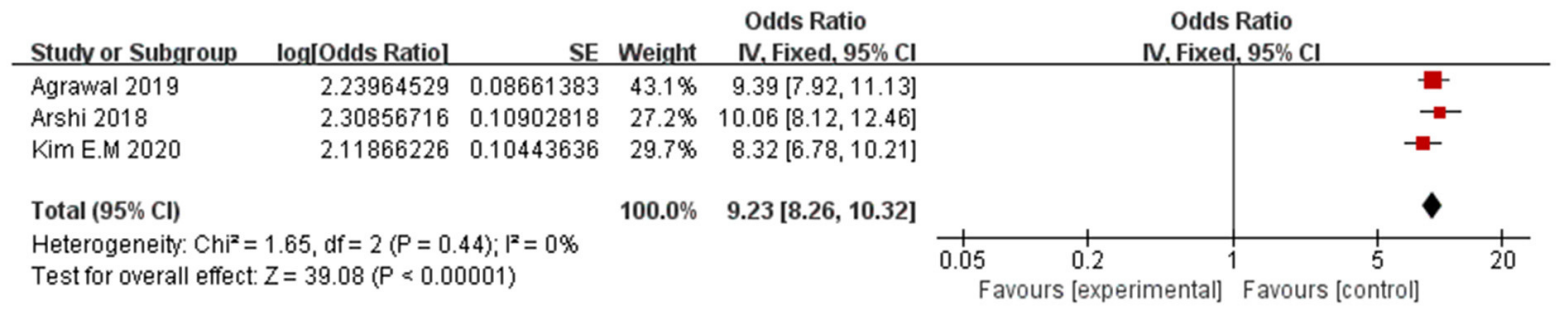
Forest plot for preoperative delirium.

Three papers (Arshi et al., 2018; Agrawal et al., 2019; Kim et al., 2020) reported preoperative delirium as a significant risk factor for POD. A fixed-effects model was applied because no significant heterogeneity was found among these studies (*P* = 0.44, I^2^ = 0%). The meta-analysis of the pooled studies suggest that preoperative delirium is a significant risk factor for the development of POD (OR: 9.23, 95% CI 8.26–10.32, *P* < 0.00001, Figure 8). This means that the incidence of POD in patients with preoperative delirium Is 9.23 times the incidence of pod in patients without preoperative delirium.

#### Diabetes

Five papers (Wang et al., 2018, 2021; He et al., 2020; Haynes et al., 2021; Ahn and Bang, 2022) reported preoperative diabetes as a significant risk factor for POD. A random-effects model was applied because significant heterogeneity was found among these studies (*P* < 0.00001, I^2^ = 87%). The meta-analysis of the pooled studies suggest that preoperative diabetes is a significant risk factor for the development of POD (OR: 1.18, 95% CI 1.05–1.33, *P* = 0.006, Additional File 3 in Supplementary material).

#### Preoperative functional dependence

Five papers (van der Zanden et al., 2016; Flikweert et al., 2018; Kim et al., 2020; Haynes et al., 2021; Jeon and Sohng, 2021) reported preoperative functional dependence as a significant risk factor for POD. A random-effects model was applied because significant heterogeneity was found among these studies (*P* = 0.008, I^2^ = 71%). The meta-analysis of the pooled studies suggests that preoperative functional dependence is a significant risk factor for the development of POD (OR: 1.31, 95% CI 1.11–1.56, *P* = 0.002, Additional File 4 in Supplementary material).

#### ASA level

Three papers (Oh et al., 2016; Arshi et al., 2018; Davani et al., 2021) reported higher ASA level (per level increase) as a significant risk factor for POD. A random-effects model was applied because significant heterogeneity was found among these studies (*P* = 0.003, I^2^ = 83%). The meta-analysis of the pooled studies suggests that higher ASA level (per level increase) is a significant risk factor for the development of POD in geriatric patients after hip fracture surgeries (OR: 1.63, 95% CI 1.04–2.57, *P* = 0.03, Additional File 5 in Supplementary material). Another 8 studies (Vochteloo et al., 2011; Kim et al., 2013, 2020; Wang et al., 2018, 2021; Zhang et al., 2019; Uzoigwe et al., 2020; Haynes et al., 2021) reported ASA level≥3 as a significant risk factor for POD. A random-effects model was applied because significant heterogeneity was found among these studies (*P* = 0.003, I^2^ = 68%). The meta-analysis of the pooled studies suggest that ASA level≥3 is a significant risk factor for the development of POD in geriatric patients after hip fracture surgeries (OR: 1.76, 95%CI 1.39–2.24, *P* < 0.00001, Additional File 6 in Supplementary material).

#### Other factors

Another 7 factors including general anesthesia, medical comorbidities, Parkinson's disease, smoking, surgery delay>48 h, longer surgical duration were analyzed. The results in Table 1 indicate that low albumin (OR: 3.30, 95% CI 1.44–7.55, *P* = 0.005), medical comorbidities (OR: 1.15, 95% CI 1.06–1.25, *P* = 0.0006), Parkinson's disease (OR: 4.17, 95% CI 1.68–10.31, *P* = 0.002) and surgery delay>48 h (OR: 1.90, 95% CI 1.36–2.65, *P* = 0.0002) are significant risk factors for POD in geriatric patients undergoing hip fracture surgeries, while general anesthesia (OR: 1.44, 95%CI 0.71–2.94, *P* = 0.32), smoking (OR: 1.80, 95% CI 0.88–3.65, *P* = 0.11), longer surgical duration (OR: 2.82, 95% CI 0.69–11.50, *P* = 0.15) are not significant risk factors.

**Table 1:** Results of meta-analysis of other factors.

**Factor**	**Number of study**	**OR**	**95% CI**	***p* value**	**I^2^ (%)**	**Test (p)**	**Statistical method**
General anesthesia	2	1.44	0.71–2.94	0.32	80	0.03	IV, Random
Longer surgical duration	2	2.82	0.69–11.50	0.15	57	0.13	IV, Random
Low albumin	2	3.30	1.44–7.55	0.005	0	0.38	IV, fixed
Medical comorbidities	2	1.15	1.06–1.25	0.0006	0	0.66	IV, fixed
Parkinson's disease	2	4.17	1.68–10.31	0.002	0	0.72	IV, fixed
Smoking	2	1.80	0.88–3.65	0.11	69	0.07	IV, Random
Surgery delay>48 h	2	1.90	1.36–2.65	0.0002	48	0.16	IV, fixed

### Sensitivity analysis

A sensitivity analysis was conducted respectively for the analysis of age (per year increase), male, preoperative dementia and ASA level≥3. We excluded each study one by one to explore whether a single study significantly impacts the heterogeneity or the results. Overall, we found that when it comes to factors including age (per year increase), male, preoperative dementia, the heterogeneity and the results were not significantly affected by any single study and that although when analyzing the ASA level≥3, after we excluding the study by Wang Y et al., the heterogeneity decreased significantly, the result changed little. Therefore, the result of our meta-analysis was relatively robust.

## Discussion

POD is prevalent among geriatric patients undergoing hip fracture surgeries. POD is a common, acute, under-recognized adverse event and is associated with significant morbidity and mortality in hospitalized elderly patients, Considering these serious complications, it is essential to recognize the risk factors of delirium to prevent it after surgery (Yang et al., 2021). Therefore we conducted this meta-analysis to investigate the potential risk factors for the occurrence of POD. Compared with previous meta-analysis (Yang et al., 2017; Wu et al., 2021), our systematic review and meta-analysis included newly-published articles in the latest 2 years and studies concerning risk factors for preoperative delirium, perioperative delirium or younger patients were excluded.

### Postoperative delirium assessment

From the results of the meta-analysis we found that the incidence of POD varies from 10.09 to 51.28%, which is similar to the former meta-analysis (Yang et al., 2017). In some institutes, the incidence of delirium is relatively high. The wide range may result from the differences including the differences in inclusion and exclusion criteria, diagnosis of POD, time and frequency of screening for POD.

The most frequently used scale is CAM. Developed by Inouye et al. based on the DSM-III for delirium, CAM has 4 remarkable features: acute onset and fluctuating course, inattention, disorganized thinking and altered level of consciousness (Inouye et al., 1990). In former studies, its sensitivities ranged from 77 to 92% and specificity ranged from 96 to 100% (Hestermann et al., 2009; Wongpakaran et al., 2011; Martins et al., 2015). Its performance in the diagnosis of delirium is excellent. However, CAM is inferior to other screening methods such as CAM-ICU and Nu-DESC in terms of time consuming. CAM-ICU and Nu-DESC can be conducted within around 5 minutes, which makes it more feasible for daily use by nurses (Han et al., 2014; Pipanmekaporn et al., 2014; Zastrow et al., 2021).

### Risk factors

The age limit of susceptibility to delirium remains controversial. From this meta-analysis, we found that when people grow older, the risk for POD is increasing year by year. For those older than 80 years old, the incidence of POD is 2.26 times that in patients younger than 80. This is comparable to previous meta-analysis (Yang et al., 2017; Wu et al., 2021). The result may be due to the fact that elderly patients were more likely influenced by age-related physical and psychical changes, such as poor organ compensative capacity, reduced body adaptability, and declined adjustment ability (van der Mast, 1998).

Patients who developed postoperative delirium were more often males. Zhu et al. (2017) deemed that women could deal with postoperative psychological stress better than male and thus was associated with less delirium. Interestingly, although in most of the included studies, female patients make up the majority, those who are markedly more likely to develop POD are male patients. Factors that may contribute to the strong association between male sex and POD include underlying disease severity, more comorbidities, and more postoperative complications (Oh et al., 2016; Oberai et al., 2021).

Preoperative cognitive impairment, as measured often by the Mini-Mental State Exam (MMSE) (Goldenberg et al., 2006; Koskderelioglu et al., 2017; Mazzola et al., 2017), has been found to be an important predictor for POD. Many studies (Liang et al., 2015; Chu et al., 2016; Yang et al., 2017) have proved the feasibility of preoperative cognitive testing in emergencies. It should become a part of the standardized program for preoperative clinical assessment for orthopedic surgeries.

Cole et al. (2009) elucidated the relationship between preoperative dementia and delirium, indicating that both had similar symptoms and pathogenesis, including metabolic rates and impaired cholinergic function, and similar causative factors, such as excitotoxic neuronal damage and neuron death (Blass and Gibson, 1999; Cole et al., 2002). Elucidation of the link between delirium and dementia could lead to the development of decided strategies for early detection, prevention and intervention strategies in patients with preoperative dementia undergoing hip fracture surgery.

For patients diagnosed with preoperative delirium, the risk of the occurrence of POD is about 10 times the risk for patients without preoperative delirium. Thus, early diagnosis and treatment of delirium is crucial to prevent the occurrence of POD.

We found a significant correlation between preoperative low albumin and POD, which appeared to indicate that a poor nutritional condition might be a potential risk factor (Lee and Park, 2010). Patients with diabetes or preoperative functional dependence also need more attention.

Our evaluation showed that the risk of POD was 1.76 times higher in patients undergoing hip surgeries with ASA≥3 than in patients with ASA <3. ASA classification is a commonly used index for pre-anesthesia risk assessment of patients formulated by the American Medical association, and the higher the rating, the worse the health status of patients (Allen, 2016). Patients with ASA ≥ 3 classification often have more serious systemic diseases and limited physical activities. The preoperative status of the patient is a key determinant of postoperative recovery. Therefore, the physical condition of a patient plays an important role in the recovery after hip surgeries.

Several previous reports compared the use of general anesthesia and regional anesthesia to reduce morbidity and mortality, including delirium. They reported that regional anesthesia yielded more favorable outcomes than general anesthesia (Mauermann et al., 2006; Guay et al., 2018). However, our meta-analysis shows that the General anesthesia is not a relevant risk factor for POD.

In our study, surgical delay was identified as a significant risk factor for POD. In previous study, Lefaivre KA.et al and Rizk.et al had proposed that, for patients with hip fracture, a surgical delay of more than 24 h was a significant predictor for POD and fast-track pathway was needed to reduce the POD incidence (Lefaivre et al., 2009; Rizk et al., 2016). Similarly, many counties had developed guidelines to support the fast-track pathway of hip surgery.

### Strengths and limitations of this meta-analysis

The present meta-analysis has strengths over the previous systematic reviews because it contains more cohort studies and more severe inclusion and exclusion criteria. To our knowledge, this is the first meta-analysis concerning the risk factors for POD in geriatric patients undergoing hip fracture surgeries, for former meta-analysis included studies of younger patients or concerning risk factors for preoperative delirium or perioperative delirium. Besides, only available odds ratio (OR) or relative risk (RR) with 95% confidence interval (95% CI) as a result of a multivariate logistic regression was extracted.

There are several limitations in this meta-analysis: (1) significant heterogeneity was found within the selected studies. (2) only articles published in English were included. (3) different assessment time and assessment scale for delirium may introduce bias. (4) for certain identified factors, only small numbers of included studies were available, and as a result, the statistical power might not be enough to detect potential associations.

## Conclusions

Based on all the relevant studies, some risk factors for POD in geriatric inpatients with hip fracture were identified. Possible significant risk factors include advance age, male, preoperative cognitive impairment, preoperative delirium, preoperative dementia, diabetes, preoperative functional dependence, high ASA level, low albumin, medical comorbidities, Parkinson's disease and surgical delay. Clinicians should be alert to patients with those factors. To identify the risk factors more precisely, more research studies with larger sample size and better design should be conducted.

## Data availability statement

The original contributions presented in the study are included in the article/Supplementary material, further inquiries can be directed to the corresponding authors.

## Author contributions

All authors listed have made a substantial, direct, and intellectual contribution to the work and approved it for publication.

## Funding

This work was supported by Winfast Charity Foundation (granted number: YL20220225).

## Conflict of interest

The authors declare that the research was conducted in the absence of any commercial or financial relationships that could be construed as a potential conflict of interest.

## Publisher's note

All claims expressed in this article are solely those of the authors and do not necessarily represent those of their affiliated organizations, or those of the publisher, the editors and the reviewers. Any product that may be evaluated in this article, or claim that may be made by its manufacturer, is not guaranteed or endorsed by the publisher.
