# Supplementary material for: Risk factors for postoperative delirium in geriatric patients with hip fracture: A systematic review and meta-analysis
Source: Front Aging Neurosci. 2022 Aug 3;14:960364. doi: 10.3389/fnagi.2022.960364 (PMC9382199; doi:10.3389/fnagi.2022.960364)
Supplement: Supplementary file 1 [file Table_1.DOCX]

**Additional File 1**

| Study | Country | Study type | Diagnosis of POD | Sample size | POD No. | Incidence of POD | Mean age  （years） | | Gender  (female/male) | |
| --- | --- | --- | --- | --- | --- | --- | --- | --- | --- | --- |
|  |  |  |  |  |  |  | POD | NPOD | POD | NPOD |
| Ahn et al.（2021） | Korean | retrospective | NM | 58972 | 8680 | 14.70% | NM | NM | NM | NM |
| Davani et al.（2021） | USA | retrospective | CAM | 566 | NM | NM | NM | NM | NM | NM |
| Haynes et al.（2021） | USA | retrospective | NM | 18754 | 3519 | 18.80% | NM | NM | NM | NM |
| Jeon et al.（2021） | Korean | retrospective | Nu-DESC | 231 | 104 | 45.02% | 83.5 | 79 | 72/32 | 90/37 |
| Oberai et al.（2021） | Australia and  New Zealand | prospective | CAM CAM-ICU3D-CAM The 4AT | 6668 | 2599 | 38.98% | 85.8 | 82.7 | 1692/904 | 2865/1207 |
| Wang et al.（2021） | China | Retrospective | CAM | 272 | 52 | 19.12% | NM | NM | 37/15 | 157/63 |
| Aldwikat et al.（2020） | Australia | Retrospective | NM | 260 | 63 | 24.23% | 86.2 | 80.7 | 42/21 | 135/62 |
| Kim E.M et al.（2020） | USA | Retrospective | NM | 6210 | 1816 | 29.24% | NM | NM | 1278/538 | 4412/1798 |
| Uzoigwe et al.（2020） | UK | Retrospective | 4AT | 1023 | 242 | 23.66% | 84.3 | 80.7 | 183/59 | 526/255 |
| He et al.（2020） | China | prospective | CAM | 780 | 182 | 23.33% | 75.77 | 73.28 | 99/83 | 302/296 |
| Cho et al.（2020） | Korean | Retrospective | CAM | 283 | 48 | 16.96% | NM | NM | NM | NM |
| Xing et al.  （2020） | China | prospective | CAM | 163 | 57 | 34.97% | 74.2 | 71.8 | 32/25 | 61/45 |
| Agrawal et al.（2019） | USA | Retrospective | NM | 8466 | 2574 | 30.40% | NM | NM | NM | NM |
| Harris et al.（2019） | USA | Retrospective | NM | 548 | 228 | 41.61% | NM | NM | 162/62 | 243/77 |
| Zhang et al.（2019） | China | Retrospective | DSM-5 | 825 | 118 | 14.30% | 79 | 79 | 85/33 | 509/198 |
| Ravi et al.（2019） | Canada | retrospective | ICD-10 | 68131 | 7150 | 10.49% | NM | NM | NM | NM |
| Arshi et al.（2018） | USA | retrospective | NM | 8439 | 2569 | 30.44% | NM | NM | 1811/758 | 4220/1650 |
| Wang et al.（2018） | China | retrospective | CAM | 306 | 59 | 19.28% | 81.9 | 76.4 | 36/23 | 166/81 |
| Flikweert et al.（2018） | Netherlands | Prospective | NM | 479 | 98 | 20.46% | NM | NM | NM | NM |
| Levinoff et al.（2018） | Canada | retrospective | CAM | 114 | 20 | 17.54% | 88.1 | 82.3 | 15/5 | 69/25 |
| Choi et al.（2017） | Korean | retrospective | CAM  CAM-ICU | 356 | 59 | 16.57% | 81.5 | NM | 51/8 | NM |
| Koskderelioglu et al.（2017） | Turkey | Prospective | CAM-ICU | 109 | 20 | 18.35% | 79.5 | 77.03 | 10/10 | 56/33 |
| Mazzola et al.（2017） | Italy | Prospective | CAM  DSM-IV | 415 | 124 | 29.88% | NM | NM | NM | NM |
| Guo et al.（2016） | China | Prospective | CAM  DSM-IV | 572 | 120 | 20.98% | 82 | 76 | 84/36 | 282/170 |
| Oh et al.（2016） | USA | Prospective | CAM | 431 | 147 | 34.11% | NM | NM | 95/52 | 220/64 |
| Shin et al.（2016） | Korean | Prospective | CAM | 78 | 40 | 51.28% | 82.8 | 80.4 | 29/11 | 34/4 |
| van der Zanden et al.（2016） | Netherlands | Prospective | DSM-IV | 415 | 135 | 32.53% | 87.9 | 83.5 | 93/42 | 206/74 |
| Zheng et al.（2016） | China | Prospective | CAM | 182 | 68 | 37.36% | 79.6 | 73.4 | 43/25 | 69/45 |
| Chen et al.（2014） | China | retrospective | CAM | 186 | 70 | 37.63% | 80.1 | 74.7 | 50/20 | 89/27 |
| Kim S.D et al.（2013） | Korean | retrospective | DSM-IV | 504 | 59 | 11.71% | NM | NM | NM | NM |
| Nie et al.（2012） | China | Prospective | DRS-R-98 | 123 | 16 | 13.01% | 75 | 75.3 | 12/4 | 73/34 |
| Lee H.B et al.（2011） | USA | Prospective | CAM,  MMSE | 425 | 149 | 35.06% | NM | NM | NM | NM |
| Vochteloo et al.（2011） | Netherlands | Prospective | DSM-IV | 1262 | 317 | 25.12% | NM | NM | NM | NM |
| Chrispal et al.（2010） | India | Prospective | CAM | 81 | 17 | 20.99% | 73.7 | 71.3 | 11/6 | 38/26 |
| Juliebø et al.（2009） | Norway | Prospective | CAM | 187 | 68 | 36.36% | 85 | 82 | 55/13 | 90/29 |
| Goldenberg et al.（2006） | USA | Prospective | CAM | 77 | 37 | 48.05% | NM | NM | NM | NM |
| Kagansky et al.（2004） | Israel | Prospective | CAM | 102 | 12 | 11.76% | 82.5 | 82.3 | 10/2 | 66/24 |

**Additional Table 1.** The general characteristic of the included studies.

**Abbreviations:**POD= postoperative delirium; NPOD= non postoperative delirium, NM= not mentioned; CAM= confusion assessment method; CAM-ICU= confusion assessment method for the intensive-care unit; DSM-IV=Diagnostic and Statistical Manual of Mental Disorders, Fourth Edition; DSM-5= Diagnostic and Statistical Manual of Mental Disorders:5th Edition; DRS-R-98= Delirium Rating Scale-Revised-98 ; ICD10= International Statistical Classification of Diseases and Related Health Problems, Tenth Revision; 4AT= 4A’s test; Nu-DESC= Nursing Delirium Screening Scale.
