# Supplementary material for: Risk factors for postoperative delirium in geriatric patients with hip fracture: A systematic review and meta-analysis
Source: Front Aging Neurosci. 2022 Aug 3;14:960364. doi: 10.3389/fnagi.2022.960364 (PMC9382199; doi:10.3389/fnagi.2022.960364)
Supplement: Supplementary file 2 [file Table_2.DOCX]

**Additional File 2**

|  | NOS scale | | | | | | | |  |
| --- | --- | --- | --- | --- | --- | --- | --- | --- | --- |
| Study | Representativeness of the exposed cohort,  1 Point | Selection of the non exposed cohort,  1 Point | Ascertainment of exposure,  1 Point | Demonstration that outcome of interest was not present at start of study,  1 Point | Comparability,2 Points | Assessment of outcome,  1 Point | Follow-up long enough for outcomes to occur,  1 Point | Adequacy of follow up of cohorts,  1 Point | Total,  9 Points |
| Ahn et al.（2021） | 1 | 1 | 1 | 1 | 2 | 1 | 1 | 0 | 8 |
| Davani et al.（2021） | 1 | 1 | 0 | 1 | 2 | 1 | 0 | 1 | 7 |
| Haynes et al.（2021） | 1 | 1 | 1 | 1 | 2 | 1 | 1 | 0 | 8 |
| Jeon et al.（2021） | 1 | 1 | 1 | 1 | 2 | 1 | 1 | 0 | 8 |
| Oberai et al.（2021） | 1 | 1 | 1 | 1 | 2 | 1 | 1 | 0 | 8 |
| Wang et al.（2021） | 1 | 1 | 1 | 1 | 2 | 1 | 0 | 0 | 7 |
| Aldwikat et al.（2020） | 1 | 1 | 1 | 1 | 2 | 1 | 1 | 0 | 8 |
| Kim E.M et al.（2020） | 1 | 1 | 1 | 1 | 2 | 1 | 1 | 0 | 8 |
| Uzoigwe et al.（2020） | 1 | 1 | 1 | 1 | 2 | 1 | 0 | 0 | 7 |
| He et al.（2020） | 1 | 1 | 0 | 1 | 2 | 1 | 1 | 0 | 7 |
| Cho et al.（2020） | 1 | 1 | 0 | 1 | 2 | 0 | 0 | 1 | 6 |
| Xing et al.  （2020） | 1 | 1 | 1 | 1 | 2 | 1 | 1 | 0 | 8 |
| Agrawal et al.（2019） | 1 | 1 | 1 | 1 | 2 | 0 | 0 | 0 | 6 |
| Harris et al.（2019） | 1 | 1 | 1 | 1 | 2 | 1 | 0 | 1 | 8 |
| Zhang et al.（2019） | 1 | 1 | 0 | 1 | 2 | 1 | 0 | 0 | 6 |
| Ravi et al.（2019） | 1 | 1 | 1 | 1 | 2 | 1 | 0 | 1 | 8 |
| Arshi et al.（2018） | 1 | 1 | 1 | 1 | 2 | 0 | 1 | 1 | 8 |
| Wang et al.（2018） | 1 | 1 | 1 | 1 | 2 | 1 | 0 | 0 | 7 |
| Flikweert et al.（2018） | 1 | 1 | 1 | 1 | 2 | 1 | 1 | 0 | 8 |
| Levinoff et al.（2018） | 1 | 1 | 1 | 1 | 2 | 0 | 0 | 0 | 6 |
| Choi et al.（2017） | 1 | 1 | 1 | 1 | 2 | 1 | 0 | 0 | 7 |
| Koskderelioglu et al.（2017） | 1 | 1 | 1 | 1 | 2 | 1 | 1 | 0 | 8 |
| Mazzola et al.（2017） | 1 | 1 | 1 | 1 | 2 | 1 | 0 | 0 | 7 |
| Guo et al.（2016） | 1 | 1 | 1 | 1 | 2 | 1 | 1 | 0 | 8 |
| Oh et al.（2016） | 1 | 1 | 1 | 1 | 2 | 1 | 0 | 0 | 7 |
| Shin et al.（2016） | 1 | 1 | 1 | 1 | 2 | 1 | 1 | 0 | 8 |
| van der Zanden et al.（2016） | 1 | 1 | 1 | 1 | 2 | 1 | 1 | 0 | 8 |
| Zheng et al.（2016） | 1 | 1 | 1 | 1 | 2 | 1 | 1 | 0 | 8 |
| Chen et al.（2014） | 1 | 1 | 1 | 1 | 2 | 1 | 1 | 0 | 8 |
| Kim S.D et al.（2013） | 1 | 1 | 1 | 1 | 2 | 1 | 0 | 0 | 7 |
| Nie et al.（2012） | 1 | 1 | 1 | 1 | 2 | 1 | 1 | 0 | 8 |
| Lee H.B et al.（2011） | 1 | 1 | 1 | 1 | 2 | 1 | 1 | 0 | 8 |
| Vochteloo et al.（2011） | 1 | 1 | 1 | 1 | 2 | 1 | 0 | 0 | 7 |
| Chrispal et al.（2010） | 1 | 1 | 1 | 1 | 2 | 1 | 0 | 0 | 7 |
| Juliebø et al.（2009） | 1 | 1 | 1 | 1 | 2 | 1 | 0 | 0 | 7 |
| Goldenberg et al.（2006） | 0 | 1 | 1 | 1 | 2 | 1 | 1 | 0 | 7 |
| Kagansky et al.（2004） | 1 | 1 | 1 | 1 | 2 | 1 | 1 | 0 | 8 |

**Additional Table 2.** The result of methodological quality assessment
