# Supplementary figures and images for: Risk factors for postoperative delirium in geriatric patients with hip fracture: A systematic review and meta-analysis
Source: Front Aging Neurosci. 2022 Aug 3;14:960364. doi: 10.3389/fnagi.2022.960364 (PMC9382199; doi:10.3389/fnagi.2022.960364)

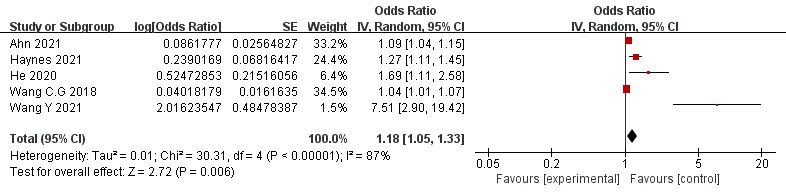

Supplement: Supplementary file 3 [file Image_1.TIFF]

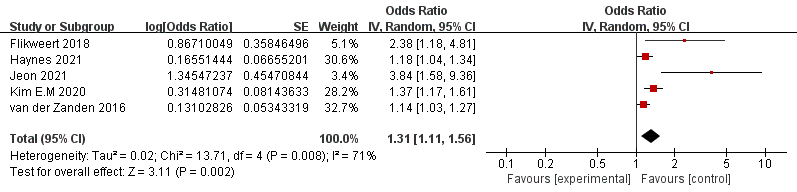

Supplement: Supplementary file 4 [file Image_2.TIFF]

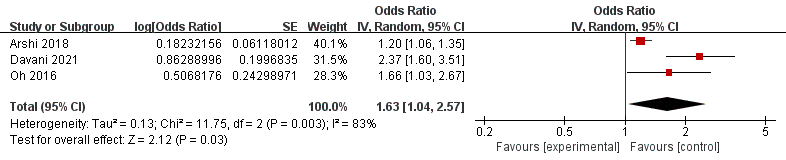

Supplement: Supplementary file 5 [file Image_3.TIFF]

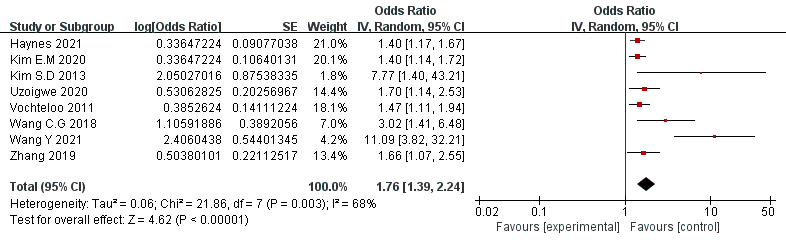

Supplement: Supplementary file 6 [file Image_4.TIFF]
